# Supplementary material for: A data-driven eXtreme gradient boosting machine learning model to predict COVID-19 transmission with meteorological drivers
Source: PLoS One. 2022 Sep 13;17(9):e0273319. doi: 10.1371/journal.pone.0273319 (PMC9469970; doi:10.1371/journal.pone.0273319)
Supplement: S1 File — (DOCX) [file pone.0273319.s003.docx]

**A data-driven eXtreme Gradient Boosting machine learning model to predict COVID-19 transmission with meteorological drivers**

We fitted the ARIMAX model for each SAARC country using all the meteorological factors listed in the main text to find the significant factors which impact the transmission of COVID-19 confirmed cases. The estimated parameters of the ARIMAX model for each country were shown in table S1.

**Table A. Estimated parameters of the fitted ARIMAX model for SAARC countries**

|  | Afghanistan | Bangladesh | Bhutan | India | Maldives | Nepal | Pakistan | Sri Lanka |
| --- | --- | --- | --- | --- | --- | --- | --- | --- |
| Parameters | ARIMAX (3,1,0) | ARIMAX(0,1,0) | ARIMAX(5,1,0) | ARIMAX(2,1,0) | ARIMAX(1,1,0) | ARIMAX(1,1,0) | ARIMAX(5,0,0) | ARIMAX(5,1,0) |
| AR(1) | -0.86 |  | -0.72 | -0.16 | -0.46 | -0.41 | 0.40 | -0.90 |
| AR(2) | -0.62 |  | -0.45 | -0.15 |  |  | 0.21 | -0.65 |
| AR(3) | -0.33 |  | -0.18 |  |  |  | 0.19 | -0.51 |
| AR(4) |  |  | -0.15 |  |  |  | 0.07 | -0.54 |
| AR(5) |  |  | -0.08 |  |  |  | 0.09 | -0.28 |

ARIMAX: Autoregressive Integrated Moving Average with Exogeneous variables

After examining the significant factors, we again fitted ARIMAX model and the XGBoost model using those significant factors as covariates. For ARIMAX model, we adjusted the power transformation parameter lambda at which the time series is stable. The estimated parameters of the ARIMAX model were shown below:

**Table B. Estimated parameters of the fitted ARIMAX model for predicting COVID-19 confirmed cases in SAARC countries**

|  | Afghanistan | Bangladesh | Bhutan | India | Maldives | Nepal | Pakistan | Sri Lanka |
| --- | --- | --- | --- | --- | --- | --- | --- | --- |
| Parameters | ARIMAX (2,1,4) | ARIMAX(0,1,00 | ARIMAX(5,1,0) | ARIMAX(4,1,0) | ARIMAX(3,1,0) | ARIMAX(1,1,0) | ARIMAX(4,1,0) | ARIMAX(5,1,0) |
| AR(1) | -0.416 |  | -0.72 | -0.45 | -0.483 | -0.41 | -0.59 | -0.58 |
| AR(2) | -0.9429 |  | -0.45 | -0.25 | 0.0092 |  | -0.37 | -0.32 |
| AR(3) |  |  | -0.18 | -0.15 | 0.1604 |  | -0.17 | -0.19 |
| AR(4) |  |  | -0.15 | -0.12 |  |  | -0.09 | -0.20 |
| AR(5) |  |  | -0.08 |  |  |  |  | -0.11 |
| MA(1) | -0.4483 |  |  |  |  |  |  |  |
| MA(2) | 0.7186 |  |  |  |  |  |  |  |
| MA(3) | -0.6629 |  |  |  |  |  |  |  |
| MA(4) | 0.1936 |  |  |  |  |  |  |  |

ARIMAX: Autoregressive Integrated Moving Average with Exogeneous variables

For XGBoost model, we also adjusted different parameters of XGBoost model for better accuracy and reducing overfitting. The adjusted parameters are listed below:

**Table C. Adjusted parameters of the XGBoost model for SAARC countries**

| Parameters | Afghanistan | Bangladesh | Bhutan | India | Maldives | Nepal | Pakistan | Sri Lanka |
| --- | --- | --- | --- | --- | --- | --- | --- | --- |
| nrounds | 10 | 1000 | 1000 | 1000 | 9 | 100 | 9 | 10 |
| nfold | 10 | 10 | 10 | 15 | 10 | 10 | 10 | 10 |
| nrounds_method | cv | cv | cv | cv | cv | cv | cv | cv |
| trend_method | none | none | none | none | none | none | none | none |
| lambda | 0.3 | 0.2 | 0.1 | 0.2 | 0.5 | 0.1 | 0.1 | 0.1 |

XGBoost: eXtreme Gradient Boosting

We trained our model using 90% of the data and made prediction in remaining 10% of the data. The point estimate of the ARIMAX and XGBoost model for SAARC countries were given below:

**Table D. Point estimate from ARIMAX and XGBoost model for Afghanistan**

| Days | ARIMAX | XGBoost |
| --- | --- | --- |
| 2021.325 | 60.69 | 52.45 |
| 2021.326 | 86.30 | 49.82 |
| 2021.327 | 101.25 | 52.26 |
| 2021.328 | 75.06 | 24.34 |
| 2021.329 | 38.51 | 31.84 |
| 2021.330 | 98.98 | 33.95 |
| 2021.331 | 83.64 | 36.11 |
| 2021.332 | 62.20 | 30.03 |
| 2021.333 | 63.95 | 19.46 |
| 2021.334 | 65.46 | 19.70 |
| 2021.335 | 59.82 | 30.05 |
| 2021.336 | 62.15 | 27.77 |
| 2021.337 | 82.72 | 43.77 |
| 2021.338 | 54.89 | 40.35 |
| 2021.339 | 45.88 | 34.29 |
| 2021.340 | 57.89 | 37.68 |
| 2021.341 | 101.78 | 25.56 |
| 2021.342 | 57.03 | 16.69 |
| 2021.343 | 91.99 | 19.26 |
| 2021.344 | 100.95 | 38.46 |
| 2021.345 | 93.83 | 34.81 |
| 2021.346 | 68.54 | 36.87 |
| 2021.347 | 68.18 | 26.00 |
| 2021.348 | 44.15 | 21.17 |
| 2021.349 | 37.56 | 29.20 |
| 2021.350 | 25.33 | 35.85 |
| 2021.351 | 74.94 | 41.81 |
| 2021.352 | 96.27 | 39.18 |
| 2021.353 | 88.16 | 40.64 |
| 2021.354 | 53.52 | 30.66 |
| 2021.355 | 41.65 | 35.01 |
| 2021.356 | 73.59 | 34.48 |
| 2021.357 | 43.03 | 42.86 |
| 2021.358 | 59.22 | 41.03 |
| 2021.359 | 60.99 | 38.41 |
| 2021.360 | 27.70 | 29.77 |
| 2021.361 | 24.83 | 24.10 |
| 2021.362 | 63.94 | 25.84 |
| 2021.363 | 67.95 | 14.71 |
| 2021.364 | 88.34 | 43.47 |
| 2021.365 | 20.86 | 57.18 |
| 2022.001 | 34.36 | 77.10 |
| 2022.002 | 25.11 | 89.31 |
| 2022.003 | 38.08 | 85.36 |
| 2022.004 | 35.84 | 63.17 |
| 2022.005 | 59.33 | 70.60 |
| 2022.006 | 45.15 | 79.59 |
| 2022.007 | 64.82 | 94.62 |
| 2022.008 | 37.06 | 112.99 |
| 2022.009 | 58.53 | 107.26 |
| 2022.010 | 80.39 | 103.82 |
| 2022.011 | 87.51 | 87.55 |
| 2022.012 | 75.93 | 66.09 |
| 2022.013 | 57.64 | 101.71 |
| 2022.014 | 58.38 | 102.51 |
| 2022.015 | 56.67 | 144.84 |
| 2022.016 | 58.66 | 128.94 |
| 2022.017 | 65.71 | 98.31 |
| 2022.018 | 59.94 | 108.05 |
| 2022.019 | 45.53 | 110.80 |
| 2022.020 | 69.99 | 101.49 |
| 2022.021 | 85.74 | 117.64 |
| 2022.022 | 54.39 | 150.47 |
| 2022.023 | 42.02 | 189.46 |
| 2022.024 | 81.67 | 115.70 |
| 2022.025 | 102.61 | 177.81 |
| 2022.026 | 68.16 | 162.94 |
| 2022.027 | 76.37 | 133.22 |
| 2022.028 | 90.97 | 162.30 |
| 2022.029 | 74.05 | 206.43 |
| 2022.030 | 71.79 | 233.27 |

**Table E. Point estimate from ARIMAX and XGBoost model for Bangladesh**

| Days | ARIMAX | XGBoost |
| --- | --- | --- |
| 2021.324 | 165.47 | 212.94 |
| 2021.325 | 120.06 | 226.29 |
| 2021.326 | 161.82 | 195.39 |
| 2021.327 | 131.02 | 192.02 |
| 2021.328 | 102.84 | 210.44 |
| 2021.329 | 75.18 | 186.02 |
| 2021.330 | 127.37 | 140.52 |
| 2021.331 | 166.52 | 185.97 |
| 2021.332 | 142.51 | 196.86 |
| 2021.333 | 172.78 | 196.92 |
| 2021.334 | 219.23 | 205.54 |
| 2021.335 | 151.90 | 209.62 |
| 2021.336 | 66.30 | 180.70 |
| 2021.337 | 99.71 | 149.67 |
| 2021.338 | 134.16 | 200.85 |
| 2021.339 | 131.02 | 220.77 |
| 2021.340 | 127.37 | 207.15 |
| 2021.341 | 128.41 | 222.56 |
| 2021.342 | 169.13 | 249.71 |
| 2021.343 | 136.77 | 188.08 |
| 2021.344 | 141.99 | 195.38 |
| 2021.345 | 131.02 | 184.10 |
| 2021.346 | 99.19 | 208.41 |
| 2021.347 | -72.02 | 260.65 |
| 2021.348 | -10.95 | 225.54 |
| 2021.349 | 105.45 | 231.53 |
| 2021.350 | 117.98 | 189.14 |
| 2021.351 | 47.51 | 178.64 |
| 2021.352 | 95.53 | 286.52 |
| 2021.353 | 45.42 | 271.15 |
| 2021.354 | 118.50 | 224.79 |
| 2021.355 | 125.80 | 211.21 |
| 2021.356 | 116.41 | 225.07 |
| 2021.357 | 111.19 | 194.19 |
| 2021.358 | 107.54 | 226.73 |
| 2021.359 | 123.20 | 189.87 |
| 2021.360 | 78.83 | 312.35 |
| 2021.361 | 96.05 | 298.77 |
| 2021.362 | 124.76 | 207.67 |
| 2021.363 | 123.72 | 249.27 |
| 2021.364 | 129.98 | 251.69 |
| 2021.365 | 152.95 | 200.14 |
| 2022.001 | 148.77 | 306.68 |
| 2022.002 | 159.21 | 281.35 |
| 2022.003 | 182.70 | 296.16 |
| 2022.004 | 149.81 | 307.70 |
| 2022.005 | 116.93 | 321.33 |
| 2022.006 | 112.76 | 238.27 |
| 2022.007 | 65.78 | 275.16 |
| 2022.008 | 98.66 | 270.06 |
| 2022.009 | 104.93 | 278.68 |
| 2022.010 | 113.28 | 292.05 |
| 2022.011 | 115.89 | 295.55 |
| 2022.012 | 112.76 | 313.11 |
| 2022.013 | 121.11 | 396.25 |
| 2022.014 | 121.63 | 182.18 |
| 2022.015 | 112.76 | 267.81 |
| 2022.016 | 157.64 | 264.45 |
| 2022.017 | 146.16 | 294.89 |
| 2022.018 | 120.59 | 332.32 |
| 2022.019 | 114.32 | 293.75 |
| 2022.020 | 135.20 | 290.12 |
| 2022.021 | 46.99 | 333.67 |
| 2022.022 | 85.09 | 337.85 |
| 2022.023 | 0.54 | 344.26 |
| 2022.024 | 60.04 | 354.85 |
| 2022.025 | 109.10 | 334.08 |
| 2022.026 | 160.78 | 365.70 |
| 2022.027 | 143.03 | 265.04 |

**Table F. Point estimate from ARIMAX and XGBoost model for Bhutan**

| Days | ARIMAX | XGBoost |
| --- | --- | --- |
| 2021.324 | 4.08 | 2.98 |
| 2021.325 | 2.83 | 2.95 |
| 2021.326 | 2.81 | 1.91 |
| 2021.327 | 3.09 | 2.82 |
| 2021.328 | 3.10 | 3.05 |
| 2021.329 | 2.76 | 2.36 |
| 2021.330 | 2.76 | 1.61 |
| 2021.331 | 2.97 | 1.35 |
| 2021.332 | 2.87 | 1.17 |
| 2021.333 | 2.58 | 1.17 |
| 2021.334 | 3.42 | 2.47 |
| 2021.335 | 2.97 | 1.30 |
| 2021.336 | 2.99 | 1.08 |
| 2021.337 | 4.15 | 1.24 |
| 2021.338 | 3.42 | 1.02 |
| 2021.339 | 4.05 | 1.02 |
| 2021.340 | 3.24 | 1.08 |
| 2021.341 | 3.97 | 1.02 |
| 2021.342 | 4.54 | 1.08 |
| 2021.343 | 3.56 | 1.02 |
| 2021.344 | 3.12 | 1.02 |
| 2021.345 | 3.60 | 1.08 |
| 2021.346 | 4.22 | 1.02 |
| 2021.347 | 4.46 | 1.02 |
| 2021.348 | 3.86 | 1.02 |
| 2021.349 | 4.60 | 1.08 |
| 2021.350 | 4.58 | 1.08 |
| 2021.351 | 4.26 | 1.02 |
| 2021.352 | 3.45 | 1.02 |
| 2021.353 | 4.03 | 1.02 |
| 2021.354 | 3.21 | 1.02 |
| 2021.355 | 3.01 | 1.02 |
| 2021.356 | 3.12 | 1.02 |
| 2021.357 | 2.97 | 1.08 |
| 2021.358 | 3.07 | 1.19 |
| 2021.359 | 2.38 | 1.19 |
| 2021.360 | 2.76 | 1.44 |
| 2021.361 | 3.54 | 2.16 |
| 2021.362 | 3.54 | 2.43 |
| 2021.363 | 3.79 | 2.32 |
| 2021.364 | 3.24 | 2.09 |

**Table G. Point estimate from ARIMAX and XGBoost model for India**

| Days | ARIMAX | XGBoost |
| --- | --- | --- |
| 2021.322 | 11229.44 | 10932.21 |
| 2021.323 | 11310.81 | 11867.49 |
| 2021.324 | 11494.32 | 10865.29 |
| 2021.325 | 11878.39 | 9491.64 |
| 2021.326 | 12036.01 | 9482.31 |
| 2021.327 | 12251.92 | 12378.11 |
| 2021.328 | 12223.68 | 12266.06 |
| 2021.329 | 12072.45 | 13661.78 |
| 2021.330 | 12346.66 | 11938.11 |
| 2021.331 | 12684.29 | 12308.32 |
| 2021.332 | 12815.77 | 10795.88 |
| 2021.333 | 12634.06 | 10937.65 |
| 2021.334 | 12678.94 | 12824.20 |
| 2021.335 | 12796.57 | 12870.96 |
| 2021.336 | 12757.52 | 12861.09 |
| 2021.337 | 12718.61 | 11975.41 |
| 2021.338 | 12707.94 | 11245.76 |
| 2021.339 | 12654.90 | 10177.74 |
| 2021.340 | 12743.25 | 12221.48 |
| 2021.341 | 12917.98 | 12356.73 |
| 2021.342 | 13167.69 | 15336.73 |
| 2021.343 | 13377.22 | 13080.08 |
| 2021.344 | 13425.41 | 11689.15 |
| 2021.345 | 13683.91 | 10922.27 |
| 2021.346 | 13839.62 | 11364.22 |
| 2021.347 | 13996.92 | 10682.95 |
| 2021.348 | 13908.46 | 12071.10 |
| 2021.349 | 13759.62 | 13009.62 |
| 2021.350 | 13793.82 | 12085.43 |
| 2021.351 | 13966.04 | 10736.09 |
| 2021.352 | 14078.04 | 10366.20 |
| 2021.353 | 14347.81 | 10225.97 |
| 2021.354 | 14638.28 | 12727.86 |
| 2021.355 | 14674.44 | 12488.02 |
| 2021.356 | 14678.45 | 12452.59 |
| 2021.357 | 14779.37 | 11203.95 |
| 2021.358 | 14880.92 | 10858.44 |
| 2021.359 | 14819.89 | 11745.86 |
| 2021.360 | 15020.02 | 11066.36 |
| 2021.361 | 15305.97 | 12212.59 |
| 2021.362 | 15784.87 | 15656.08 |
| 2021.363 | 16048.95 | 15693.64 |
| 2021.364 | 16299.30 | 16885.19 |
| 2021.365 | 16392.40 | 13746.62 |
| 2022.001 | 16237.44 | 13902.24 |
| 2022.002 | 16294.82 | 12831.89 |
| 2022.003 | 16692.66 | 15251.62 |
| 2022.004 | 16733.36 | 19053.33 |
| 2022.005 | 16184.56 | 17139.90 |
| 2022.006 | 16224.14 | 18476.45 |
| 2022.007 | 16494.82 | 15096.87 |
| 2022.008 | 17062.24 | 18534.23 |
| 2022.009 | 17382.92 | 14591.62 |
| 2022.010 | 17766.28 | 15768.22 |
| 2022.011 | 17924.63 | 18800.87 |
| 2022.012 | 18045.42 | 19693.98 |
| 2022.013 | 18580.77 | 20229.68 |
| 2022.014 | 18526.08 | 16730.95 |
| 2022.015 | 18690.51 | 17711.65 |
| 2022.016 | 18896.62 | 18400.01 |
| 2022.017 | 19084.38 | 16758.86 |
| 2022.018 | 19315.01 | 22428.99 |
| 2022.019 | 19053.78 | 20843.13 |
| 2022.020 | 18876.34 | 20916.48 |
| 2022.021 | 18841.02 | 21595.05 |
| 2022.022 | 19356.19 | 23229.44 |
| 2022.023 | 19884.00 | 20323.82 |
| 2022.024 | 20774.20 | 19585.20 |
| 2022.025 | 21112.27 | 22664.98 |
| 2022.026 | 21728.72 | 23309.80 |

**Table H. Point estimate from ARIMAX and XGBoost model for Maldives**

| Days | ARIMAX | XGBoost |
| --- | --- | --- |
| 2021.324 | 105.60 | 97.16 |
| 2021.325 | 93.17 | 95.14 |
| 2021.326 | 94.63 | 101.93 |
| 2021.327 | 94.68 | 108.32 |
| 2021.328 | 95.63 | 133.59 |
| 2021.329 | 97.54 | 112.38 |
| 2021.330 | 96.30 | 128.60 |
| 2021.331 | 98.34 | 119.69 |
| 2021.332 | 98.61 | 123.69 |
| 2021.333 | 96.82 | 120.38 |
| 2021.334 | 95.32 | 110.92 |
| 2021.335 | 97.17 | 116.19 |
| 2021.336 | 98.66 | 111.89 |
| 2021.337 | 96.21 | 122.98 |
| 2021.338 | 100.07 | 112.35 |
| 2021.339 | 105.62 | 105.26 |
| 2021.340 | 107.83 | 97.72 |
| 2021.341 | 103.34 | 96.66 |
| 2021.342 | 103.41 | 103.69 |
| 2021.343 | 97.12 | 101.81 |
| 2021.344 | 105.62 | 109.05 |
| 2021.345 | 114.73 | 109.96 |
| 2021.346 | 111.13 | 115.80 |
| 2021.347 | 114.70 | 88.48 |
| 2021.348 | 109.77 | 94.02 |
| 2021.349 | 105.21 | 108.16 |
| 2021.350 | 106.23 | 93.74 |
| 2021.351 | 109.70 | 98.59 |
| 2021.352 | 113.37 | 99.78 |
| 2021.353 | 112.08 | 102.30 |
| 2021.354 | 107.90 | 105.03 |
| 2021.355 | 112.08 | 105.27 |
| 2021.356 | 109.84 | 126.60 |
| 2021.357 | 114.70 | 137.74 |
| 2021.358 | 115.68 | 138.25 |
| 2021.359 | 99.97 | 133.49 |
| 2021.360 | 102.56 | 107.24 |
| 2021.361 | 109.46 | 122.69 |
| 2021.362 | 107.45 | 128.87 |
| 2021.363 | 108.61 | 142.44 |
| 2021.364 | 108.37 | 115.02 |
| 2021.365 | 106.30 | 114.07 |
| 2022.001 | 111.33 | 119.06 |
| 2022.002 | 111.71 | 119.06 |
| 2022.003 | 102.90 | 105.99 |
| 2022.004 | 102.83 | 110.52 |
| 2022.005 | 103.44 | 122.62 |
| 2022.006 | 103.34 | 118.35 |
| 2022.007 | 107.08 | 109.98 |
| 2022.008 | 104.29 | 119.06 |
| 2022.009 | 102.49 | 124.60 |
| 2022.010 | 105.69 | 120.65 |
| 2022.011 | 109.36 | 116.39 |
| 2022.012 | 109.53 | 119.56 |
| 2022.013 | 112.05 | 136.69 |
| 2022.014 | 108.51 | 128.27 |
| 2022.015 | 108.37 | 128.00 |
| 2022.016 | 110.69 | 131.36 |
| 2022.017 | 110.07 | 113.10 |
| 2022.018 | 109.22 | 113.10 |
| 2022.019 | 108.07 | 117.18 |
| 2022.020 | 105.65 | 129.59 |
| 2022.021 | 107.35 | 138.90 |
| 2022.022 | 114.63 | 119.45 |
| 2022.023 | 112.18 | 124.12 |
| 2022.024 | 114.90 | 105.10 |
| 2022.025 | 112.18 | 136.11 |
| 2022.026 | 111.20 | 136.69 |
| 2022.027 | 101.03 | 119.45 |
| 2022.028 | 89.77 | 105.71 |

**Table I. Point estimate from ARIMAX and XGBoost model for Nepal**

| Days | ARIMAX | XGBoost |
| --- | --- | --- |
| 2021.322 | 366.52 | 725.23 |
| 2021.323 | 368.04 | 694.55 |
| 2021.324 | 338.28 | 736.57 |
| 2021.325 | 309.80 | 613.85 |
| 2021.326 | 309.40 | 686.44 |
| 2021.327 | 348.75 | 640.07 |
| 2021.328 | 344.82 | 621.13 |
| 2021.329 | 362.46 | 669.37 |
| 2021.330 | 303.17 | 750.50 |
| 2021.331 | 364.73 | 748.47 |
| 2021.332 | 386.70 | 597.88 |
| 2021.333 | 371.80 | 668.21 |
| 2021.334 | 319.07 | 603.83 |
| 2021.335 | 340.51 | 639.51 |
| 2021.336 | 320.32 | 648.10 |
| 2021.337 | 390.50 | 799.49 |
| 2021.338 | 372.33 | 572.15 |
| 2021.339 | 342.29 | 685.45 |
| 2021.340 | 256.20 | 648.34 |
| 2021.341 | 294.32 | 619.40 |
| 2021.342 | 293.07 | 572.25 |
| 2021.343 | 345.03 | 640.45 |
| 2021.344 | 362.72 | 645.00 |
| 2021.345 | 406.12 | 650.81 |
| 2021.346 | 410.69 | 601.66 |
| 2021.347 | 341.52 | 580.59 |
| 2021.348 | 262.78 | 587.01 |
| 2021.349 | 326.14 | 585.75 |
| 2021.350 | 346.32 | 594.75 |
| 2021.351 | 296.58 | 554.30 |
| 2021.352 | 359.45 | 410.40 |
| 2021.353 | 392.52 | 367.51 |
| 2021.354 | 403.86 | 472.26 |
| 2021.355 | 364.98 | 436.30 |
| 2021.356 | 358.20 | 439.75 |
| 2021.357 | 380.12 | 413.90 |
| 2021.358 | 406.37 | 335.26 |
| 2021.359 | 423.77 | 339.62 |
| 2021.360 | 435.64 | 291.78 |
| 2021.361 | 453.57 | 300.76 |
| 2021.362 | 472.75 | 242.58 |
| 2021.363 | 352.38 | 331.87 |
| 2021.364 | 412.18 | 337.89 |
| 2021.365 | 384.68 | 345.77 |
| 2022.001 | 378.63 | 324.84 |
| 2022.002 | 333.69 | 277.44 |
| 2022.003 | 346.32 | 197.18 |
| 2022.004 | 287.78 | 134.77 |
| 2022.005 | 256.20 | 129.43 |
| 2022.006 | 239.08 | 170.66 |
| 2022.007 | 244.09 | 146.28 |
| 2022.008 | 264.80 | 114.95 |
| 2022.009 | 216.35 | 103.56 |
| 2022.010 | 227.21 | 102.64 |
| 2022.011 | 310.71 | 101.23 |
| 2022.012 | 306.67 | 96.16 |
| 2022.013 | 329.41 | 99.31 |
| 2022.014 | 364.98 | 97.51 |
| 2022.015 | 332.92 | 103.41 |
| 2022.016 | 289.80 | 83.97 |
| 2022.017 | 237.79 | 119.12 |
| 2022.018 | 279.70 | 77.76 |
| 2022.019 | 282.45 | 76.70 |
| 2022.020 | 306.19 | 85.14 |
| 2022.021 | 308.69 | 98.83 |
| 2022.022 | 329.16 | 115.51 |
| 2022.023 | 315.80 | 93.09 |
| 2022.024 | 280.43 | 83.26 |
| 2022.025 | 378.10 | 80.39 |
| 2022.026 | 329.41 | 73.86 |
| 2022.027 | 374.83 | 104.33 |
| 2022.028 | 348.59 | 88.97 |
| 2022.029 | 301.14 | 82.71 |
| 2022.030 | 293.07 | 103.35 |

**Table J. Point estimate from ARIMAX and XGBoost model for Pakistan**

| Days | ARIMAX | XGBoost |
| --- | --- | --- |
| 2021.325 | 323.01 | 369.73 |
| 2021.326 | 329.99 | 372.11 |
| 2021.327 | 311.52 | 411.17 |
| 2021.328 | 312.02 | 411.17 |
| 2021.329 | 304.25 | 411.17 |
| 2021.330 | 301.34 | 381.41 |
| 2021.331 | 318.89 | 411.17 |
| 2021.332 | 331.58 | 411.17 |
| 2021.333 | 318.68 | 372.11 |
| 2021.334 | 315.52 | 372.11 |
| 2021.335 | 325.78 | 372.11 |
| 2021.336 | 329.00 | 372.11 |
| 2021.337 | 325.75 | 372.11 |
| 2021.338 | 327.37 | 372.11 |
| 2021.339 | 318.21 | 411.17 |
| 2021.340 | 315.51 | 411.17 |
| 2021.341 | 319.82 | 411.17 |
| 2021.342 | 323.06 | 411.17 |
| 2021.343 | 320.36 | 411.17 |
| 2021.344 | 323.60 | 411.17 |
| 2021.345 | 331.15 | 411.17 |
| 2021.346 | 331.15 | 365.97 |
| 2021.347 | 331.15 | 365.97 |
| 2021.348 | 323.06 | 411.17 |
| 2021.349 | 328.46 | 411.17 |
| 2021.350 | 334.93 | 411.17 |
| 2021.351 | 321.98 | 365.97 |
| 2021.352 | 320.36 | 411.17 |
| 2021.353 | 333.85 | 429.66 |
| 2021.354 | 332.77 | 381.41 |
| 2021.355 | 320.90 | 411.17 |
| 2021.356 | 324.14 | 411.17 |
| 2021.357 | 325.22 | 411.17 |
| 2021.358 | 313.35 | 429.66 |
| 2021.359 | 306.88 | 429.66 |
| 2021.360 | 314.43 | 411.17 |
| 2021.361 | 316.05 | 411.17 |
| 2021.362 | 313.35 | 429.66 |
| 2021.363 | 313.89 | 411.17 |
| 2021.364 | 319.28 | 429.66 |
| 2021.365 | 324.68 | 410.01 |
| 2022.001 | 312.27 | 373.47 |
| 2022.002 | 310.65 | 299.42 |
| 2022.003 | 312.81 | 353.70 |
| 2022.004 | 303.10 | 429.66 |
| 2022.005 | 299.86 | 389.02 |
| 2022.006 | 313.89 | 429.66 |
| 2022.007 | 316.59 | 429.66 |
| 2022.008 | 322.52 | 373.47 |
| 2022.009 | 325.76 | 391.54 |
| 2022.010 | 323.60 | 429.66 |
| 2022.011 | 317.13 | 389.02 |
| 2022.012 | 306.34 | 389.02 |
| 2022.013 | 307.96 | 389.02 |
| 2022.014 | 313.35 | 371.05 |
| 2022.015 | 317.67 | 337.65 |
| 2022.016 | 319.82 | 333.00 |
| 2022.017 | 333.31 | 391.54 |
| 2022.018 | 337.63 | 429.66 |
| 2022.019 | 332.23 | 303.86 |
| 2022.020 | 329.00 | 240.94 |
| 2022.021 | 325.76 | 240.94 |
| 2022.022 | 317.67 | 325.30 |
| 2022.023 | 315.51 | 265.66 |
| 2022.024 | 312.81 | 278.01 |
| 2022.025 | 302.02 | 352.91 |
| 2022.026 | 298.24 | 371.05 |
| 2022.027 | 306.34 | 295.64 |
| 2022.028 | 308.49 | 295.64 |
| 2022.029 | 317.13 | 299.82 |

**Table K. Point estimate from ARIMAX and XGBoost model for Sri Lanka**

| Days | ARIMAX | XGBoost |
| --- | --- | --- |
| 2021.322 | 783.97 | 582.47 |
| 2021.323 | 848.56 | 571.80 |
| 2021.324 | 805.69 | 525.29 |
| 2021.325 | 789.82 | 620.90 |
| 2021.326 | 757.58 | 525.29 |
| 2021.327 | 741.35 | 521.75 |
| 2021.328 | 769.27 | 525.29 |
| 2021.329 | 795.29 | 592.11 |
| 2021.330 | 798.53 | 592.11 |
| 2021.331 | 772.26 | 474.80 |
| 2021.332 | 764.29 | 488.76 |
| 2021.333 | 783.98 | 445.42 |
| 2021.334 | 757.53 | 445.93 |
| 2021.335 | 739.62 | 447.50 |
| 2021.336 | 764.62 | 565.20 |
| 2021.337 | 800.64 | 442.03 |
| 2021.338 | 803.99 | 445.97 |
| 2021.339 | 790.02 | 445.97 |
| 2021.340 | 784.90 | 445.97 |
| 2021.341 | 774.66 | 473.70 |
| 2021.342 | 786.65 | 512.99 |
| 2021.343 | 784.94 | 445.97 |
| 2021.344 | 798.75 | 415.97 |
| 2021.345 | 825.13 | 461.31 |
| 2021.346 | 828.69 | 378.37 |
| 2021.347 | 818.04 | 378.37 |
| 2021.348 | 816.27 | 417.84 |
| 2021.349 | 826.92 | 461.31 |
| 2021.350 | 834.06 | 445.97 |
| 2021.351 | 830.48 | 430.38 |
| 2021.352 | 823.36 | 430.38 |
| 2021.353 | 828.70 | 415.97 |
| 2021.354 | 821.58 | 415.97 |
| 2021.355 | 837.65 | 430.38 |
| 2021.356 | 848.49 | 445.97 |
| 2021.357 | 835.85 | 445.97 |
| 2021.358 | 830.48 | 445.97 |
| 2021.359 | 821.58 | 445.97 |
| 2021.360 | 814.51 | 445.97 |
| 2021.361 | 826.91 | 445.97 |
| 2021.362 | 841.25 | 445.97 |
| 2021.363 | 857.59 | 393.90 |
| 2021.364 | 841.25 | 393.90 |
| 2021.365 | 863.08 | 393.90 |
| 2022.001 | 866.76 | 393.90 |
| 2022.002 | 876.00 | 393.90 |
| 2022.003 | 859.42 | 373.58 |
| 2022.004 | 852.12 | 373.58 |
| 2022.005 | 857.59 | 367.07 |
| 2022.006 | 864.92 | 367.07 |
| 2022.007 | 883.44 | 415.97 |
| 2022.008 | 883.44 | 415.97 |
| 2022.009 | 866.76 | 415.97 |
| 2022.010 | 846.67 | 415.97 |
| 2022.011 | 852.12 | 415.97 |
| 2022.012 | 859.42 | 415.97 |
| 2022.013 | 864.92 | 430.38 |
| 2022.014 | 850.30 | 430.38 |
| 2022.015 | 846.67 | 415.97 |
| 2022.016 | 857.59 | 415.97 |
| 2022.017 | 853.94 | 415.97 |
| 2022.018 | 843.06 | 491.20 |
| 2022.019 | 852.12 | 385.64 |
| 2022.020 | 857.59 | 372.58 |
| 2022.021 | 835.85 | 430.38 |
| 2022.022 | 832.27 | 415.97 |
| 2022.023 | 830.48 | 430.38 |
| 2022.024 | 826.91 | 378.37 |
| 2022.025 | 823.36 | 390.81 |
| 2022.026 | 821.58 | 390.81 |
| 2022.027 | 830.48 | 390.81 |
| 2022.028 | 846.67 | 430.38 |
| 2022.029 | 870.45 | 390.81 |
| 2022.030 | 870.45 | 390.81 |

**Table L. Estimated Error measures of the ARIMAX and XGBoost model.**

| Country | ARIMAX | | | | XGBoost | | | |
| --- | --- | --- | --- | --- | --- | --- | --- | --- |
|  | RMSE | MAE | MPE | MAPE | RMSE | MAE | MPE | MAPE |
| Afghanistan | 81.87 | 52.46 | 150.74 | 174.44 | 67.53 | 43.18 | 123.77 | 148.36 |
| Bangladesh | 5438.13 | 2772.52 | 72.13 | 72.13 | 5333.29 | 2644.12 | 48.46 | 51.35 |
| Bhutan | 82.05 | 52.59 | 16.26 | 98 | 81.27 | 51.48 | 44.52 | 89.98 |
| India | 157149.6 | 92470.54 | 2.39 | 73.97 | 156978.7 | 91973.83 | 12.49 | 66.5 |
| Maldives | 1100.99 | 602.65 | 43.07 | 50 | 1093.29 | 595.78 | 38.92 | 48.97 |
| Nepal | 3147.78 | 1568.51 | 7.13 | 66.15 | 243.4 | 202.86 | 51.99 | 57.65 |
| Pakistan | 3254.68 | 1871.86 | 40.58 | 49.66 | 3240.45 | 1855.86 | 26.51 | 52.13 |
| Sri Lanka | 373.6 | 220.43 | 22.26 | 31.48 | 455.69 | 333.61 | 40.73 | 40.93 |
| Average | 21328.59 | 12451.45 | 44.32 | 76.98 | 20936.70 | 12212.59 | 48.42 | 69.48 |

ARIMAX: Autoregressive Integrated Moving Average with Exogeneous variable; XGBoost: eXtreme Gradient Boosting; RMSE: Root Mean Square Error; MAE: Mean Absolute Error; MPE: Mean Percentage Error; MAPE: Mean Absolute Percentage Error.
