# Supplementary material for: A data-driven eXtreme gradient boosting machine learning model to predict COVID-19 transmission with meteorological drivers
Source: PLoS One. 2022 Sep 13;17(9):e0273319. doi: 10.1371/journal.pone.0273319 (PMC9469970; doi:10.1371/journal.pone.0273319)
Supplement: Attachment — Submitted filename: Response to Reviewers.docx [file pone.0273319.s004.docx]

**PLOS ONE**

**PONE-D-22-14088**

**Identification of significant meteorological risk factors and accuracy comparison of ARIMAX and XGBoost models for COVID-19 prediction in SAARC Countries**

**Reviewers' comments:**

Reviewer #1: This study found that meteorological factors had both positive and negative impacts on the transmission of COVID-19 confirmed cases. However, to conclude this, the study needs a rigorous regression analysis to control other essential covariates (demographic, socioeconomic, healthcare, human mobility, etc.).

**Comment:** We are a bit at a loss on how to adjust the comment. This includes a separate objective and needs a completely separate analysis. However, we added the limitation of our study incorporating the comments (please see lines 353-362).

In Table 1, some significant values at the 5% level are inconsistent with the confidence intervals. If a confidence interval contains zero, there is strong evidence that there is no 'significant' difference between the two population means.

**Comment:** We corrected the mistake in Table 2 and updated the result section accordingly (please Table 2, lines 262-273).

There are typographical or grammatical errors, including the agent's name for COVID-19. And finally, the authors need to explain their research findings as the evidence to date is inconsistent.

**Comment:** We made corrections throughout the whole manuscript for any typographical or grammatical errors. We also updated the introduction ( please see lines 59-64, 78-83, 86-90, 93-96, 101-102), discussion (lines 290-303, 321-328) conclusion section (lines 357-364).

Reviewer #2: Title is not so attractive and easily understandable. Abbreviation need to be avoided from the title. The tile is large enough, so please concise and make it attractive for laymen reader also.

**Comment:** Thanks for your suggestion. We updated the topic of the title accordingly. Some method-based abbreviations are popular and widely used.

Abstract- Methodology part need to be more elaborately mentioned, how data was analysis need to be mentioned. The conclusion section is more general rather need to mention about what specific action need to be taken.

**Comment:** Thanks for your suggestion. We updated the methodology section and conclusion section (please see lines 357-364).

Introduction- Section is very short, how climate is related to COVID-19 need to be more emphasized in introduction.

**Comment:** We added texts updated the introduction section accordingly (please see 59-64, 78-83, 86-90, 93-96, 101-102).

Methodology- Though this is a secondary source of data, but not clearly mentioned. How and from where the permission was taken from the data utilization need to be mentioned. What are the parameters use for the study need to be added. Ethical the ethical permission was taken for this study.

**Comment:** Thanks for your comment. Since we used freely available data, we don’t need to take permission from any source. However, we added the Ethics approval and consent to participate section in our manuscript.

Result- Interesting findings. But why country wise the result is different can be a matter of fact. In Afghanistan and Pakistan the transmission of COVID-19 cases negatively impact with minimum temperature, but reverse in other two countries but why, is there any other factors. Need to measure again the degree of error in your study findings. The findings could not equally achieve all three objective you mentioned.

**Comment:** We updated the result section and calculated the average across the various training/testing splits shown (please see Supplementary file 3). We also updated the discussion section (please see lines 290-303, 321-328) conclusion section (lines 357-364).

Conclusion- Is not so attractive, need rewrite ignoring general sentence in such special innovative research. What we can do in future should be directed in the conclusion.

**Comment:** We added texts and updated the conclusion section (please see lines 357-364).

Reviewer #3: The study aimed at identifying significant meteorological risk factors and accuracy comparison of ARIMAX and XGBoost models for COVID-19 prediction in SAARC Countries. The study was well designed. They used sophisticated machine learning algorithms for doing so and from my expertise I think that they have been executed accurately. The results were presented in a well-organized manner. The findings are well-grounded and will be very helpful for the scientific community working the field of COVID-19. Therefore, I am recommending this study for publication.

**Comment:** Thanks.

Reviewer #4: The paper is interesting, however, the key contribution is not clear. Please add additional paragraph of theoretical / practical significance of the research. Also, please copy-edit the entire manuscript. The references are not followed proper guideline:

For example:

175 29. Hyndman RJ. AG. Forecasting: principles and practice. [cited 28 Feb 2022]. Available:

176 https://books.google.com.bd/books?hl=en&lr=&id=_bBhDwAAQBAJ&oi=fnd&pg=PA7

177 &dq=Forecasting+principles+and+practice&ots=Tii-

178 tiZPFJ&sig=Q6Y58Rd860QSTBEi1BxeriFw4Z0&redir_esc=y#v=onepage&q=Forecastin

179 g principles and practice&f=false

180 30. Franses PH. Primary Demand for Beer in The Netherlands : An Application of ARMAX

181 Model. 1991;xxvm: 240–245.

182 31. Lv CX, An SY, Qiao BJ, Wu W. Time series analysis of hemorrhagic fever with renal

183 syndrome in mainland China by using an XGBoost forecasting model. BMC Infect Dis.

184 2021;21: 1–13. doi:10.1186/S12879-021-06503-Y/TABLES/5

**Comment:** Thanks for your suggestion. We edited the references and updated introduction ( please see lines 59-64, 78-83, 86-90, 93-96, 101-102), discussion (please see lines 290-303, 321-328) conclusion section (lines 357-364).We have also made corrections in the text wherever we have found typos or unclear statements in the whole manuscript.
