# Supplementary material for: A data-driven eXtreme gradient boosting machine learning model to predict COVID-19 transmission with meteorological drivers
Source: PLoS One. 2022 Sep 13;17(9):e0273319. doi: 10.1371/journal.pone.0273319 (PMC9469970; doi:10.1371/journal.pone.0273319)
Supplement: Attachment — Submitted filename: Response to Reviewers.docx [file pone.0273319.s005.docx]

**PLOS ONE**

**PONE-D-22-14088**

**Identification of significant meteorological risk factors and accuracy comparison of ARIMAX and XGBoost models for COVID-19 prediction in SAARC Countries**

**Reviewers' comments:**

Reviewer #1: The key findings of this study are based on Table 2, which is not valid and not reliable with the description. The authors failed to interpret the findings in Table 2. Most of the beta values are statistically insignificant but described as significant findings in the manuscript. There is a close relationship between confidence intervals and significance tests. Specifically, if a statistic is significantly different from 0 at the 0.05 level, then the 95% confidence interval will not contain 0.

**Comment:** We renalysed and updated the result section (please see Table 2, lines 221-229, 237-242, 249-261, 278-297, 312-315).

Reviewer #2: Title need to be changed for making more attractive. The abbreviation used in the title need to remove. Three objectives are more in a article which might confuse the reader. The objective need to make easy to understand. How study finings guide the authorities need to mention in conclusion of abstract. The methodology is relatively complex to understand. Conclusion should include some points of recommendation

**Comment:** Thanks for your suggestion. We changed the title, updated the objectives in conclusion of abstract, methodology section and conclusion section (please see lines 23-30, 37-44, 46-48, 112-140, 390-394).

Reviewer #4: The author (s) tried to investigate the Time series prediction of COVID-19 transmission in SAARC countries using a datadriven XGBoost machine learning model. The revised version is good.

**Comment:** Thanks.
